# Smart Drugs “As Common As Coffee”: Media Hype about Neuroenhancement

**DOI:** 10.1371/journal.pone.0028416

**Published:** 2011-11-30

**Authors:** Bradley J. Partridge, Stephanie K. Bell, Jayne C. Lucke, Sarah Yeates, Wayne D. Hall

**Affiliations:** UQ Centre for Clinical Research, The University of Queensland, Herston, Queensland, Australia; Yale University School of Medicine, United States of America

## Abstract

**Background:**

The use of prescription drugs to improve cognitive functioning in normal persons –“neuroenhancement” – has gained recent attention from bioethicists and neuroscientists. Enthusiasts claim that the practice is widespread and increasing, and has many potential benefits; however recent evidence provides weak support for these claims. In this study we explored how the newsprint media portrays neuroenhancement.

**Aims:**

We conducted an empirical study of media reporting of neuroenhancement to explore: media portrayals of the prevalence of neuroenhancement; the types of evidence used by the media to support claims about its prevalence; and, the possible benefits and risks of neuroenhancement mentioned in these media articles.

**Methods:**

Using the Factiva database, we found 142 newspaper articles about the non-medical use prescription drugs for neuroenhancement for the period 2008-2010. We conducted a thematic content analysis of how articles portrayed the prevalence of neuroenhancement; what type of evidence they used in support; and, the potential benefits and risks/side-effects of neuroenhancement that were mentioned.

**Results:**

87% of media articles mentioned the prevalence of neuroenhancement, and 94% portrayed it as common, increasing or both. 66% referred to the academic literature to support these claims and 44% either named an author or a journal. 95% of articles mentioned at least one possible benefit of using prescription drugs for neuroenhancement, but only 58% mentioned any risks/side effects. 15% questioned the evidence for efficacy of prescription drugs to produce benefits to users.

**Conclusions:**

News media articles mentioned the possible benefits of using drugs for neuroenhancement more than the potential risks/side effects, and the main source for media claims that neuroenhancement is common and increasingly widespread has been reports from the academic literature that provide weak support for this claim. We urge journalists and researchers to be cautious in their portrayal of the non-medical use of drugs for neuroenhancement.

## Introduction

Overly enthusiastic media coverage of applications of neuroscience research can unrealistically raise public expectations about their future impact for good and ill [1], [2]. Researchers have expressed concerns that media reporting often misconstrues the scope, feasibility, benefits and risks of new neurotechnologies. For example, analyses of news reports of fMRI, Deep Brain Stimulation, and neurosurgery suggest that their coverage has exaggerated their efficacy in ways that may profoundly impact public understanding [2]. The media has been similarly accused of perpetuating “genohype” by inflating the benefits (and downplaying the risks) of genetic research [3].

The source of media exaggeration can be researchers themselves. In their examination of how the media reports the neuroscience research on Attention Deficit Hyperactivity Disorder (ADHD), Gonon and colleagues [4] found that the media often accurately reported researchers' optimistic extrapolations of preliminary data to “therapeutic prospects”, and researchers reported stronger conclusions than their results warranted. Because misleading media accounts may undermine responsible public debate it is important to understand how the media portrays scientific neuroscience research and innovation [5].

### The potential use of drugs to enhance cognition: “smart drugs”

New insights into behaviour, cognition, and mental illness afforded by the “neuroscience revolution” have increased public interest in novel applications of this research, such as the use of drugs to improve cognitive functioning in normal persons. Recent neuroscience research, for example, has raised the possibility that some prescription drugs used to treat ADHD, narcolepsy and Alzheimer's disease, may improve cognitive functions in healthy people, such as executive function, alertness, concentration and memory. This use of these drugs has been called “neuroenhancement”, or “cognitive enhancement”. The most commonly purportedly neuroenhancers [6] include modafinil (Provigil or Nuvigil) which is indicated for the treatment of narcolepsy; psychostimulants that are used to treat ADHD, such as methylphenidate (Ritalin), or mixed amphetamine salts including dextroamphetamine (Adderall); and drugs such as donepezil that are used to treat Alzheimer's Disease [7].

In a recent study of the print media's portrayal of the non-medical use of methylphenidate, Forlini and Racine [8] found enthusiasm for the use of methylphenidate as a neuroenhancer. The drug was often described in sensationalist terms as “*brain steroids*”, and “*smart pills*”. News articles also exaggerated the prevalence of neuroenhancement by describing it as widespread, running the risk of normalizing such use and encouraging others to engage in it. For example, a recent study of deaths from unintentional prescription opioid poisonings found that media articles about the nonmedical use of these drugs often preceded increases in overdose deaths [9]. Some countries have developed guidelines that promote the responsible media coverage of drug-related material in an attempt to limit the unintended indirect encouragement of nonmedical drug use (e.g. The Australian Press Council [10]). However, these guidelines focus on reporting illicit drug use rather than the enhancement use of prescription drugs.

Forlini and Racine [8] also found that the bioethics discourse on the enhancement use of methylphenidate exhibited the same enthusiasm displayed by the media. Several recent high profile articles by bioethicists and neuroscientists in leading science journals have argued that the neuroenhancement use of stimulant drugs has much to offer healthy individuals and society (e.g. [11]). They have also claimed that the enhancement use of these prescription drugs is common and increasingly prevalent, particularly among healthy university students (e.g. [6], [11], [12], [13]). Chatterjee [12], for example, has said that “*based on the belief that these drugs improve test performance, the use of stimulant medications among college students in the US is widespread*”. Another bioethics article reflecting this sentiment [6] prompted the journal *Nature* to conduct an on line poll of its readers about their use of prescription drugs for neuroenhancement – 20% of the 1400 self-selected readers who responded to the survey reported that they had used these drugs to improve their concentration [14]. Some articles have also advocated policies and professional guidelines to facilitate the practice [7] and “*help society accept the benefits of enhancement, given appropriate research and evolved regulation*” [11].

However, other researchers have urged more caution about the use of pharmaceutical drugs for neuroenhancement [15], [16], [17]. They have argued that the evidence for the effectiveness of such drugs in healthy people is questionable and that the risks of addiction and other long term dangers have not been well studied. They point out those who claim that neuroenhancement is widespread have often mistakenly equated all non-medical use of stimulants with neuroenhancement, highlighted atypically high prevalence estimates, and inflated estimated use by reporting “lifetime use” of stimulants rather than “past year” or “past month” use (for a more comprehensive discussion of this, see [17]). A recent review of 25 surveys of the prevalence of “smart drugs” usage found large variations in sample size, scope and ways in which participants were asked about how and why they used prescription stimulants non-medically [18]. These variations severely limit the conclusions that can be drawn. Larger, more representative studies typically report a past year prevalence of nonmedical stimulant of between 3–6%. Surveys that reported the highest prevalence rates tended to have smaller samples drawn from single institutions. The enhancement use of Ritalin and Adderall among US college students has also been uncritically claimed to be globally representative of college students [11]. However, a recent survey of German university students and pupils' use of prescription stimulants for neuroenhancement found that only 0.26% reported doing so in the past year [19].

Forlini and Racine [8] also noted that the enthusiasm shown in both the media and bioethics discourse was supported by very weak evidence of effectiveness (see also [20]). Several recent studies have found that the neuroenhancing effects of a number of prescription drugs in normal persons are equivocal at best [18] and systematic reviews have found that enthusiasm for the neuroenhancement use of anti-dementia drugs, methylphenidate, and modafinil “*exceeded their actual effects*” [21], [22].

### The current study

We updated and extended Forlini and Racine's analysis of 20 newspaper articles about the non-medical use of methylphenidate from the period 2000–2006 [8] to take account of the publication of a number of high-profile, often-cited articles published in high impact journals since 2007 (for example [6], [11], [14]). We accordingly conducted an empirical study of media reporting of cognitive enhancement for the period 2008–2010. Newsprint articles that discussed the use of prescription drugs (Ritalin, Adderall and modafinil) specifically for neuroenhancement purposes (rather than other non-medical uses such as recreation) were analysed. In particular, we explored: media portrayals of the prevalence of neuroenhancement; the types of evidence used by the media to support claims about the prevalence of neuroenhancement; and, the possible benefits and risks of neuroenhancement mentioned in these media articles.

## Methods

### Search strategy

We conducted a search of print newspaper articles about neuroenhancement listed in the *Factiva* database in English-speaking sources between 1^st^ January 2008 and 31^st^ December 2010. *Factiva* is widely used for comprehensive newspaper article searching and offers comparable coverage to other news service databases (e.g. *Lexis-Nexis*). We chose newspapers because they are the most widely circulated form of print media and they cater to a broad, lay readership. Our sample was generated using a number of iterative keyword searches that expanded on the search terms used by Forlini and Racine [8] by including many of the terms that their study identified in media reporting of neuroenhancement (e.g. “smart drug”, methylphenidate, Ritalin, cognitive enhancement). The search also included key terms used in the academic literature such as “cosmetic neurology” or “brain doping” and the names of drugs that are referred to as potential neuroenhancers, including their brand names (e.g. Ritalin, Provigil, Adderall). The final search strategy was:

neuroenhance* OR neuro-enhance* OR nootropic* OR cosmetic neurology OR cosmetic psychopharmacology

OR

(Ritalin OR methylphenidate OR modafinil or Adderall or dexamphetamine) AND (enhance OR enhancement OR enhancer* OR enhancing OR cognition-enhancing OR cognitive-enhanc*)

OR

(memory-boost* OR professional's pill OR professor's little helper OR smart fix OR smarts in a bottle OR smart drugs OR brain doping OR brave new brain OR clever pills OR iq-profin) AND (enhance OR enhancement OR enhancer* OR enhancing OR cognition-enhancing OR cognitive-enhanc* OR neuroenhance* OR neuro-enhance* OR nootropic* OR cosmetic neurology OR cosmetic psychopharmacology)

All articles were read and checked for relevance according to pre-established inclusion and exclusion criteria. Articles were only included if they directly discussed neuroenhancement, which we defined as the use of pharmaceutical drugs to improve cognitive function in those without a disorder. We excluded articles whose main focus was the use of stimulants for therapeutic purposes (e.g. the treatment of ADHD); enhancement use in sport; cosmetic surgery; enhancement using natural remedies; drug misuse that was not for enhancement purposes; and articles of less than 50 words; book reviews; and, duplicate articles. When articles were reprinted in shorter and longer forms both versions were considered as distinct and separate, and included in the analysis. A total of 142 articles were included in the analysis.

## Analysis

### Phase 1: Prevalence of neuroenhancement

Two members of the research team (BP and JL) independently classified each media article according to its overall portrayal of the prevalence of neuroenhancement using the following categories:

1. Neuroenhancement is common, widespread or high in prevalence (an excerpt indicative of this category was “*The survey focused on Ritalin and Modafinil. Both medications are common on college campuses as study aids*”);
2. Uncommon or low prevalence (for example “*There has always been a small but determined minority...*”);
3. Unknown prevalence (an indicative description being “*No one knows how many people are using modafinil off- label as a cognitive enhancer to improve their thinking ability*”);
4. Neuroenhancement is increasing in prevalence (an indicative description being “*Students are increasingly taking drugs like Ritalin instead of Red Bull or triple espressos*”);
5. No mention of the prevalence.

Early in the analysis we found that some articles described neuroenhancement as both “common” and “increasing” so we amended the classification structure to note these examples. After separately classifying each article, the initial agreement rate between the two researchers was 82% (116/142 articles). The two raters met to discuss the remaining 26 articles and reached a consensus on the rating.

### Phase 2: Evidence cited for prevalence

Each article in the sample was then examined by two members of the research team (BP and SB) to identify the type of evidence that was used to support claims about the prevalence of neuroenhancement. For each article, SB used NVivo 9 to code each portion of text that described the prevalence of neuroenhancement. Coded text ranged from individual sentences to entire paragraphs. Some articles made several separate claims about the prevalence of neuroenhancement whereas other articles made only one claim. Each claim was classified according to the evidence cited:

1. Anecdotal evidence (e.g. “*Michael, 23, said about a quarter of his postgraduate classmates were taking amphetamine-based stimulants to enhance their ability to study*”);
2. Academic research/studies/papers;
3. Scientist or researcher opinion/quotation;
4. Other expert opinion/quotation (e.g. physicians, university representatives);
5. No evidence.

SB and BP then reached consensus on any items that were unclear. At this point the coding structure was refined to more accurately describe how the media articles referred to “academic research/studies/papers”. Some media articles referred to specific surveys or papers from the academic literature either by naming the author(s) or journal (e.g. it was clear that the following referred to the *Nature* paper by Maher, 2008: “*The scientific journal Nature published the results of an online survey of 1,400 adults. It showed that 20% of readers had taken "smart drugs"*). Other media articles referred only to unnamed “research” or “studies” (e.g. “*One in six university students uses 'smart drugs', studies in the US show”*). We separated this code into:

1. Named academic research/ studies/papers (either by naming the journal or author)
2. Unspecified research/studies/papers.

BP then separately coded the portion of text in each article that referred to the prevalence of neuroenhancement as: 1) anecdotal evidence; 2) named academic research/studies/papers; 3) unspecified research/studies/papers; 4) scientist or researcher opinion/quotation; 5) other expert opinion/quotation (e.g. physicians, university representatives); 6) no evidence.

### Phase 3: Benefits of using prescription drugs for neuroenhancement

BP and SB conducted a thematic content analysis of the possible benefits of prescription drugs for neuroenhancement mentioned in each media article. Categories were initially derived from the most commonly noted benefits in the academic literature, and other benefits emerged during the coding process (e.g. improved attention, concentration, alertness, and memory; enhanced ability to study; improved exam performance/better grades; increase intelligence; help to stay awake/reduce fatigue; relieve jet-lag; improve learning; work faster/be more productive). Coded text claimed that prescription drugs have benefits for healthy people (for example the following sentence was coded as “improve memory” and “improve attention”: “[Ritalin] *is also effective in the healthy brain, where it improves memory and the ability to focus attention*”), or claimed that healthy people use them to achieve certain benefits (for example the following sentence was coded as “improve concentration”: “*The majority of these med-taking brainiacs said they indulged in order to improve concentration).* We also coded text that cited self-reported or purported/possible benefits, for example the following excerpt was coded as “improve exam performance/better grades” and “improve concentration”:

*Matt, a business finance student at the University of Florida, claimed a similar drug, Adderall, had helped him improve his grades. "It's a miracle drug," he told The Boston Globe. "It is unbelievable how my concentration boosts when I use it."*

During the coding process, we found that a number of articles mentioned non-specific cognitive benefits of using prescription drugs that could be described as an improvement in brain or mental functioning, for example the potential to “*fine-tune mental performance*”, or “*boost brain power*”. We created a separate code to record these instances of general, non-specific cognitive benefits. We aggregated the number of media articles in the sample that mentioned each benefit.

### Phase 4: Risks and side effects

BP and SB conducted the same type of thematic content analysis for the health risks/side-effects of using prescription drugs for neuroenhancement. Categories were derived from the most commonly mentioned risks/side-effects in the literature for example, insomnia; the potential for addiction/dependence; abuse; mental health problems including anxiety; headache; heart problems; loss of appetite/nausea. Other risks were allowed to emerge from the data. During the coding process we created a new category for texts that mentioned the possibility of non-specific risks, side effects, or dangers for example: “*drugs, especially when they are not used as intended, can have dangerous side effects. Cerebral candy should not be sold out of gumball machines”.* We also recorded the number of articles that did not mention any specific or non-specific risks, side-effects or dangers. We then aggregated the number of media articles in the sample that mentioned each risk.

## Results

### 1) Prevalence of neuroenhancement

Eighty-seven percent of media articles (124/142) made some mention of the prevalence of neuroenhancement. The other 18 focused solely on other issues. The overwhelming majority of the former (94%, 116/124) described neuroenhancement as common, increasing in prevalence, or both (see Table 1).

**Table 1:** Media portrayal of the prevalence of neuroenhancement.

DESCRIPTION OF PREVALENCE	TOTAL
Common, widespread or high prevalence & Increasing in prevalence	54^a^
Common, widespread or high prevalence	39
Increasing in prevalence	23
Did not mention prevalence	18
Low prevalence or uncommon	6
Prevalence is unknown	2

a 10 articles also included one “counter view” (6 =  low prevalence; 4 =  unknown).

Only 6 articles said that neuroenhancement was uncommon and 2 articles said that the prevalence was unclear because of a lack of empirical evidence or study limitations, for example:

*Volkow says no one knows how many people are using modafinil off- label as a "cognitive enhancer" to improve their thinking ability and work for hours on end. "It's not like anyone has done a proper survey to actually document that," Volkow says.*

Media articles varied in the number of claims they made about the prevalence of neuroenhancement – while some made only one claim, others made three or more. 10 articles that said neuroenhancement was common also included a “counter view” – that is, a statement that queried the claims made in the rest of the article without representing the dominant view in the article. These counter views (low = 6, and unclear prevalence = 4) were presented as opinion, cursorily mentioned; they were not expressed strongly enough to change the overall impression created by the article that neuroenhancement stimulant use was common.

**Neuroenhancement is common and increasing.** Ninety-three articles portrayed neuroenhancement as common. They said, for example, that “*many students*” use stimulants to improve their academic performance, that it is “*popular*”, “*commonplace*”, “*widespread*”, or even “*rife*”. The high prevalence of neuroenhancement was sometimes reflected in article titles such as “*Poll finds most take 'smart drugs' to help with studies”*; “*One in five admit using brain drugs*”; and, “*Student ‘dex’ rife*”. An article titled “*Brainiac drug use at work*” claimed that:

[Ritalin and Provigil] *are common currency on US college campuses, used as ‘study aids’ to sharpen performance and wakefulness.*

Another theme was that these drugs were being used on a regular basis, for example:

*Ashley is one of thousands of students who regularly turn to cognitive-enhancing drugs to improve their mental alertness and performance.*

The paradigm use of prescription stimulants was as a “*study aid*” by university students but widespread neuroenhancement was also reported among academics and scientists, military personnel, shift workers, doctors and other “*high-flying professionals*”. One article titled “*They call it the professional's pill*”, said that the use of smart drugs is “*spreading across all sectors of society”*.

Seventy-seven media articles portrayed the phenomenon as increasing in prevalence. It was, for example, said to be engaged in by “*more and more students worldwide*”, had “*proliferated in recent years*”, use had “*soared*”, and that “*students are increasingly taking drugs like Ritalin instead of Red Bull or triple espressos”*. Some predicted that this trajectory of use would make drugs for neuroenhancement *"as common as coffee within a decade or two”.* Other articles reported that the demand for cognitive enhancing drugs had created a “*booming internet black market”.*

### 2) Evidence cited for the prevalence of neuroenhancement

Media articles typically cited several main sources of evidence for the view that neuroenhancement was common and increasing (see Table 2). Forty articles (33%) included at least one claim about the prevalence of neuroenhancement without referring to any specific evidence for that claim, for example “*Increasing numbers of stressed students are taking 'smart pills' to boost exam performance*”. Nineteen articles (15%) included at least one claim that was based on anecdotal reports of widespread use, for example:

**Table 2:** Evidence cited by media articles for claims about the prevalence of neuroenhancement.

EVIDENCE CITED	TOTAL^a^n = 124(%)^b^
**No evidence cited for claim**	40 (33%)^c^
**Anecdotal evidence**	19 (15%)
**Academic research/study/paper**	82 (66%)
**Named research/study/paper**	55 (44%)
Maher (2008)	28 (23%)
Sahakian & Morein-Zamir (2007)	7 (6%)
Greely et al. (2008)	6 (5%)
Cakic (2009)	5 (4%)
Academy of Medical Sciences (2008)	5 (4%)
British Medical Association (2007)	3 (2%)
White et al. (2006)	2 (2%)
Other papers	4 (3%)
**Unspecified research/study/paper**	45 (36%)
**Researcher opinion**	23 (19%)
Sahakian	9 (7%)
Cakic	8 (6%)
Other researcher	6 (5%)
**Other opinion**	9 (7%)

a the total sample minus those articles that did not mention the prevalence of neuroenhancement.

b percentages are not cumulative because more than one source of evidence may be cited in each article.

c percentages have been rounded to the nearest whole number.

*“This stuff is being passed around all the time,” says one male A-level student with something of a smart-drug habit – ‘this stuff’ largely being Ritalin.*

**Unspecified research.** Eighty-two (66%) articles made at least one claim that referred to research. Forty-five articles (36%) did not give specific details about the author(s) or the journal in which it was published, simply referring to “experts”, “scientists”, “studies” or “research”. Thirty of these articles offered a prevalence rate from unspecified surveys or studies but the specific figures cited point to the probable source for some of them. These examples over-extended the findings, or reported an outlying finding from a single study. Nine articles suggested that “16%” of students use drugs for neuroenhancement, for example:

*Surveys in the United States indicate that 16% of university students are using "smart drugs".*

These media articles are most likely referring to a study by Babcock and Byrne [23] that has often been mistakenly cited in leading articles in the bioethics literature as evidence that neuroenhancement is widespread (for examples see [17]). Babcock and Byrne in fact reported that 16.6% of students in *one* liberal arts college had used Ritalin for *fun* at least once in their lives [23].

Other media articles in this category cited the findings of scientific studies more accurately but nonetheless exaggerated the prevalence of cognitive enhancement. For example:

*...prescription-only drugs are already increasingly being used to enhance memory, attention span and wakefulness. Studies show that up to a quarter of students at some U.S. universities have used them in the past year...*

The 15 media articles that mentioned a prevalence rate of 25% probably referred to McCabe et al.'s [24] study of non-medical use of prescription stimulants in 119 US colleges (which has also been widely cited in same leading bioethics articles for example Greely et al. [11]). In that study, *one* college out of 119 reported that 25% of students had used stimulants in the past year. The more representative rate of use in the past year for the whole 119 colleges was only 3% (the median). Only five articles mentioned the past year or lifetime prevalence rate in that study (4–7%) in addition to the 25% outlier.

**Specified papers from the literature.** The academic sources that the media cited as evidence for the prevalence of neuroenhancement were written by bioethicists or neuroscientists; they were rarely primary surveys of the prevalence of neuroenhancement. The most highly cited of these articles were an *online poll* in *Nature* by Maher [14] titled “Poll results: look who's doping”. This paper was mentioned by 28 media articles; a commentary in *Nature* by Sahakian and Morein-Zamir [6] titled “Professor's little helper”, mentioned by 7 articles; a commentary in *Nature* by Greely et al. [11] (6 articles); a commentary in *Journal of Medical Ethics* by Cakic [13] (5 articles), a report by the Academy of Medical Sciences [25] (5 articles); a report by the British Medical Association [26] (3 articles); and a study by Prudhomme White et al. [27] (2 articles).

A typical media reference to the Nature poll by Maher [14] was as follows:

*A poll by scientific journal Nature found one in five respondents said they took drugs such as Ritalin, and most of the 1400 respondents said adults should be allowed to use them.*

In some cases these poll results were misinterpreted as applying to all scientists, for example:

*One-fifth of the world's professional scientists and university science students have used "cognition-enhancing" prescription drugs to help them concentrate...*

Lay readers are unlikely to appreciate that a self-selected group of participants in an online poll are not representative of the “world's professional scientists”. A typical media reference to the *Nature* article by Sahakian and Morein-Zamir [6] was:

[*Stimulants*] *are popular among academics, students and workers looking for a brain boost. "Off-label and nonprescription use by the general public is becoming increasingly commonplace" Cambridge University researchers reported in the science journal Nature.*

A typical reference to Greely et al.[11] was:

*A group of experts is arguing, in an essay in the prestigious scientific journal "Nature," that we should acknowledge the fact that a growing number of healthy college students and adults are using mind-enhancing drugs to improve their performance.*

The articles by Greely et al.[11], Sahakian and Morein-Zamir [6], and Cakic [13] focused largely on ethical issues raised by cognitive enhancement. They did not report primary research or comprehensively review the literature although they did claim that neuroenhancement is widespread or increasing. One media report misreported Cakic [13] as primary evidence that:

*Research from the University of Sydney has warned that students are increasingly using `̀performance enhancing'' psychostimulants usually prescribed for Alzheimer's, Parkinson's or sleep disorders, to enhance their academic performance.*

**Researcher/expert opinion.** A small number of articles quoted experts such as university spokespeople, student representatives, or health professionals (n = 9). Twenty-three articles reported the opinion of academic researchers on the prevalence of cognitive enhancement, but only Sahakian (n = 9) and Cakic (n = 8) were quoted by more than one article. For example, one article said:

*Vince Cakic, a researcher from the University of Sydney's school of psychology, said students were increasingly using psychostimulants — drugs usually prescribed for neuropsychiatric disorders and sleep disorders — to enhance their academic performance.*

One media article reported anecdotal evidence cited by Sahakian as evidence that many academics use modafinil to enhance productivity, for example:

*Professor Sahakian said. "I went to a meeting in Florida and I said to a colleague 'I'm suffering from jetlag', and he said to me 'would you like some of my modafinil?' A lot of my colleagues (are) using it."*

**An evidential circle.** We found one interesting interaction between media and academia portrayals of the prevalence of neuroenhancement. In early 2009, the Cambridge student newspaper *Varsity* ran several articles about the use of “smart drugs” by students and academics. It then conducted its own survey of 1000 Cambridge students and reported that “*One in ten takes drugs to study*” without prescription. This poll was then reported in 3 media articles in our sample, for instance:

*A recent survey of 1,000 Cambridge undergraduates showed that one in 10 had used cognitive-enhancing drugs - also known as "professor's little helpers". Another third said they would use them if they had access to them.*

This excerpt did not indicate that the survey of Cambridge undergraduates was an informal poll conducted by a student newspaper, rather than a scientific survey published in the peer-reviewed literature. Lay readers could not discern this from the text (especially given the reference to Cambridge). The results of the *Varsity* poll have since been cited in the scientific literature [28] as evidence that cognitive enhancement use of stimulants is widespread.

### 3) Benefits of healthy people using prescription drugs for neuroenhancement

Ninety-five percent of articles listed at least one potential neuroenhancing benefit of using methylphenidate, dexamphetamine or modafinil. The most commonly mentioned were a general improvement in brain or mental functioning (for example “*improve mental prowess*”; “*boost brain power*”); improved concentration; better grades or improved exam/academic performance; improved memory; stay awake/reduce fatigue; increased alertness; and use to help study (see Table 3).

**Table 3:** Benefits of using prescription drugs for neuroenhancement mentioned in print media.

BENEFITS	TOTAL n = 142(%)a b
No benefits mentioned	7 (5%)
Improve brain/mental function	59 (42%)
Improve concentration	57 (40%)
Better exam/academic performance, or better grades	55 (39%)
Improve memory	44 (31%)
Stay awake/reduce fatigue	43 (30%)
Increase alertness	34 (24%)
Help study	30 (21%)
Improve attention	23 (16%)
Efficacy of drugs for CE is uncertain	22 (15%)
Work faster/more productive	17 (12%)
Relieve jet-lag	17 (12%)
Increase intelligence	13 (9%)
Better than caffeine	10 (7%)
Enhance learning	9 (6%)
Increase motivation	8 (6%)

a percentages are not cumulative because more than one benefit may be cited by each media article.

b percentages have been rounded to the nearest whole number.

These media articles either asserted that these drugs produced the benefits that were mentioned (e.g. “[Ritalin] *is also effective in the healthy brain, where it improves memory and the ability to focus attention*”), mentioned their possible benefits without questioning their efficacy, or implied their efficacy (e.g. “*Writing in the journal Nature, the academics argue that the prescription- only drugs are already increasingly being used to enhance memory, attention span and wakefulness.”*).

Twenty-two articles (15%) expressed some doubts about or questioned the enhancement efficacy of prescription drugs, for example:

*...proper studies on the use of enhancers in healthy people may well show a significant placebo effect, where people take a pill and then make sure they do the work. "There is considerable individual variation," he* [Dr. Sandberg] *said.*

### 4) Risks of using prescription drugs for neuroenhancement

58% of articles mentioned at least one possible health risk/side-effect of using prescription drugs for neuroenhancement (see Table 4). Most often the risks were unspecified (n = 44, 31%) for example:

**Table 4:** Risks/side-effects of using prescription drugs for neuroenhancement mentioned in print media.

RISKS/SIDE EFFECTS	TOTAL n = 142(*%*)a b
No risks/side effects mentioned	60 (42%)
Unspecified risks/side effects	44 (31%)
Mental health problems (inc. anxiety and depression)	27 (19%)
Addiction/dependency	22 (15%)
Heart or blood pressure problems	22 (15%)
Insomnia	20 (14%)
Little is known about the risks	20 (14%)
Headache	19 (13%)
Abuse	17 (12%)
Loss of appetite or nausea	16 (11%)
May be fatal	10 (7%)

a percentages are not cumulative because more than one risk/side-effect may be cited by each media article.

b percentages have been rounded to the nearest whole number.

*She* [*Barbara Sahakian*] *warns against the potential dangers of people under 20, whose brains are still developing, taking smart drugs like Ritalin unless they have a condition such as ADHD.*

33/44 articles that mentioned general risks or dangers also mentioned at least one specific risk. For example, the above-mentioned article went on to say that there is also “*a potential for abuse*”. The most commonly mentioned specific risks/side-effects in the sample were mental health problems; addiction/dependency; heart or blood pressure problems; insomnia; headache; abuse; loss of appetite or nausea; and death.

## Discussion

Our analysis indicates that most newspaper articles portray neuroenhancement as common or increasing in prevalence. Very few portrayed it as rare or of unknown prevalence. This is despite the largest and most representative surveys to date suggesting a past year prevalence of nonmedical stimulant use of 3–6% [17], [18]. Two-thirds of media articles referred to the academic literature in some way to support claims that neuroenhancement is common or increasing and 44% either named an author or a journal. The *Nature* poll by Maher [14] was the most cited paper about prevalence even though this reported an informal poll of self-selected readers that was unlikely to be representative of the journal's readership or the world's scientists. Sahakian & Morein-Zamir [6]; Greely et al. [11]; and Cakic [13], were the next most cited papers about prevalence. These were commentaries on the ethics of neuroenhancement that also claimed neuroenhancement was widespread or increasing. However they sometimes cited research in ways that misinterpreted the prevalence of neuroenhancement (see [17]).

When media articles cited prevalence estimates from the academic literature they often misinterpreted the data (e.g. reporting the finding that 20% of a self-selected sample of *Nature* readers indicated they had used drugs for cognitive enhancement as showing that “*20% of the world's scientists*”). They also presented data in ways that amplified the prevalence of neuroenhancement (e.g. by highlighting the minority of studies with higher estimated prevalence of use between 16–35%). They also failed to report important qualifications on the data (e.g. small samples from single colleges).

Interestingly, a recent review of the evidence for the prevalence of neuroenhancement alluded to increased media attention, saying “*To judge from recent reports in the popular media, healthy people have also begun to use MPH and AMPs for cognitive enhancement*” [18]. It went on to say that a number of major newspapers and other popular media “*have reported a trend toward growing use of prescription stimulants by healthy people for the purpose of enhancing school or work performance*” [18]. Our results indicate that the main source for media claims that neuroenhancement is common and increasingly widespread has been enthusiastic reports from the academic literature. As Bubela and Caulfield [3] suggest, journalists are not always the sole source of exaggerated claims about science. As with the creation of “genohype”, neuroscientists and bioethicists have contributed to the creation of a “bubble of enthusiasm” for neuroenhancement.

Media articles also provided positive statements about the enhancement benefits of using these drugs. Nearly all articles in our study (95%) mentioned at least one possible benefit of using prescription drugs for neuroenhancement, but only 58% mentioned any risks/side effects. Only a minority of articles expressed doubts about or questioned the evidence for efficacy of prescription drugs to produce benefits to users – this is despite recent reviews indicating only modest evidence for their cognitive enhancing effects [22]. This is in line with other studies showing that the media often under-reports risks whilst emphasising the potential benefits of both neuroscience innovations [1] and prescription medication [29], [30].

These findings have several implications of concern. Firstly, misleading media reporting increases the likelihood that public policy with be poorly informed. If neuroenhancers are believed to be widespread and effective for improving concentration or getting better grades, then policies may mistakenly be developed to facilitate such use. In the bioethics literature, these claims have promoted recommendations to: make it legal for students to use psychostimulants for neuroenhancement without a prescription; allow pharmaceutical companies to market these drugs to healthy people for neuroenhancement; and ensure that these drugs are available to all students so that none miss out on the advantages that they provide [11]. For example, the American Academy of Neurology has suggested that it is morally and legally acceptable for neurologists to prescribe these drugs to healthy people for the purposes on neuroenhancement [7]. Other authors have speculated about the implementation of “drug-testing” students before exams to avoid what is seen as “cheating” [13].

A second concern is about the possible impact of media reporting of neuroenhancement on reader behaviour. Forlini and Racine [31] found that healthcare providers, students and parents all viewed the media as an important source of information about neuroenhancement. Their interviewees were concerned that unbalanced reporting could unwittingly normalise this behaviour. More research is required to explore this potential effect of media coverage but there was some suggestive evidence of an effect in several media articles in our sample. These reported the views of students who implied that media reports provided the initial impetus to experiment with purported neuroenhancers. For example:

*"I read an article in the student press on them," says Lawrence Price, a third-year arts student at Sheffield Hallam University. "It was criticising them, but I thought they sounded great." Perera, similarly, found out about smart drugs through the media. "I read an article in Nature on them," he says. "They seemed a pretty good idea."*

We found that even though many newspaper articles mentioned the potential risks, most nevertheless pointed out many possible benefits. The benefits of neuroenhancement clearly stood out to the first student quoted above, even in an article that was apparently critical of the practice.

One possibility worth discussing is the potential role of media guidelines on responsible reporting of drug use. The Australian Press Council, for example, has guidelines on the responsible media coverage of illicit drug use. This limits details of reporting about the quantity, methods of administration and drug composition in an attempt to limit unintended, indirect encouragement of drug use [10]. These guidelines could be adapted to reporting of nonmedical use of prescription drugs for any purpose, including neuroenhancement. Reporters should be encouraged to cite high quality evidence (i.e. well conducted primary research studies) as the basis for claims of prevalence. They should avoid overstating benefits in ways that may encourage use.

We did not assess whether all the articles in our sample would comply with these guidelines but articles that overstate the prevalence, efficacy, and benefits of prescription drugs for neuroenhancement and not giving equal consideration to their risks arguably encourage readers to try these drugs. We found a small number of media articles that described journalists' own experimentation with prescription drugs as neuroenhancers. For example, one article described how to obtain modafinil:

*Finally, I tried a randomly picked doctor. Fortunately, his standards weren't as high as my friend's, and he gave me a prescription for a £5 'admin fee'.*

The journalist went on to describe the neuroenhancement benefits he experienced from several 100 mg and 200 mg doses, as well as other unexpected benefits such as “being the life of the party”:

*Concentration and output sky-rocketed and I felt fantastic. In fact, during my two weeks of Modafinil-enhanced life I did tons of good work, gained two new regular slots in newspapers, lost 4lb in weight and cleared an Augean stable of admin. But I'm not sure Modafinil really increases your memory or makes you more intelligent. It makes you think you're more intelligent.*

*The next morning, I had a horrendous hangover but was cheered by reports from my girlfriend, who said her friends thought I was the life and soul of the party. Was that a euphemism for loudmouth buffoon, I asked? 'No, really, you were fun', she insisted.*

### Limitations

Our findings should be interpreted with the following limitations in mind. Firstly, the generation of our media sample relied upon a search strategy consisting of key terms related to cognitive enhancement. However, the broadness of terminology used in neuroenhancement discussions also means that there may be media articles that our search terms did not capture (“false negatives”) that other search strategies may [32]. Secondly, our search strategy focused on news print media articles given that they are the most widely circulated form of print media catering to a broad, lay readership. It would be worthwhile for future investigations to explore other media such as television, magazines, radio, film, or internet blogs. Thirdly, our sample was limited to English language articles (primarily from the USA, UK and Australia) listed in the *Factiva* database.

### Conclusions

While it is easy for researchers to blame the media for misrepresenting their research, researchers also have an ethical obligation to present information in an accurate manner that is least amenable to such misrepresentation. Poor reporting, citation and translation of data all increase the potential for research to be inaccurately interpreted by other researchers and by the media [4], [33]. When important pieces of information that qualify or contextualise the results are omitted, research findings can be seriously misrepresented, as we argue has happened in the case of putative neuroenhancement use of prescribed stimulant drugs. Neuroscience and bioethics researchers, and science journal editors, have an obligation to ensure that they do not contribute to uncritical media portrayals that inflate the prevalence of cognitive enhancement, as our data suggests some have done to date.
